# Site-specific nanomodulator capable of modulation apoptosis for enhanced colorectal cancer chemo-photothermal therapy

**DOI:** 10.1186/s12951-023-01779-5

**Published:** 2023-01-20

**Authors:** Shuqi Wang, Li Zhou, Hailong Tian, Bowen Li, Miao Su, Qiong Li, Edouard C. Nice, Canhua Huang, Jichun Shao, Tao He

**Affiliations:** 1 Institute for Cancer Medicine, School of Basic Medical Sciences, Southwest Medical University, Luzhou, 646000 Sichuan China; 2 State Key Laboratory of Biotherapy and Cancer Center, West China Hospital, and West China, School of Basic Medical Sciences and Forensic Medicine, Collaborative Innovation Center for Biotherapy, Sichuan University, Chengdu, 610041 China; 3 Department of Biochemistry and Molecular Biology, Monash University, Clayton, VIC 3800 Australia; 4 The Second Affiliated Hospital of Chengdu Medical College, China National Nuclear Corporation 416 Hospital, Chengdu, 610051 Sichuan China; 5 Key Laboratory of Molecular Biology for Infectious Diseases (Ministry of Education), Institute for Viral Hepatitis, Department of Infectious Diseases, the Second Affiliated Hospital, Chongqing Medical University, Chongqing, 400016 China

**Keywords:** Colorectal cancer, Celastrol, IR820, Chemotherapy, Photothermal therapy

## Abstract

**Background:**

Colorectal cancer (CRC) is a common malignancy with the second highest mortality and the third highest morbidity worldwide. However, the overall survival of patients is unsatisfactory, thus requiring more effective clinical strategies. Celastrol (CLT), a natural bioactive compound, has been reported to induce reactive oxygen species (ROS)-mediated apoptosis to exhibit significant antitumor effects against CRC. However, the poor water solubility, low targeting ability, and bioavailability of CLT have limited its application, and CLT-induced protective autophagy weakens its therapeutic efficiency.

**Results:**

We designed a targeted chemo-phototherapy nanoplatform (HCR NPs) to improve the application of CLT. The codelivery of IR820 and CLT in HCR NPs solved the water-soluble problem of CLT and enhanced apoptosis via IR820-mediated hyperthermia. In addition, hydroxychloroquine (HCQ) conjugated to hyaluronic acid (HA) not only increased the active targeting of HCR NPs but also inhibited CLT-induced protective autophagy to exacerbate apoptosis, thus achieving an amplified antitumor effect. Importantly, the HCR NPs exhibited an excellent therapeutic effect on CRC both in vitro and in vivo.

**Conclusion:**

The HCR NPs presented in this study may not merely provide a new reference for the clinical application of CLT but also result in an attractive strategy for CRC treatment.

**Supplementary Information:**

The online version contains supplementary material available at 10.1186/s12951-023-01779-5.

## Introduction

The incidence of colorectal cancer (CRC) has increased rapidly worldwide. According to the latest data released by the American Institute for Cancer Research (AICR) and the WHO International Agency for Research on Cancer (IARC), CRC has the third highest incidence of cancer morbidity and the second highest incidence of cancer mortality in the world [1, 2]. Surgical resection is currently the main therapeutic option for CRC, but surgery alone or chemotherapy cannot effectively improve the treatment effect and survival of patients, especially those with late-stage disease. However, multimodal combination therapy is showing new prospects for clinical application, and can overcome some of the drawbacks of monotherapy while further improving anticancer efficiency by different therapeutic mechanisms [3, 4]. Therefore, developing new therapeutic strategies and drugs is urgently needed.

Celastrol (CLT), isolated from the root extracts of the vine Tripterygium wilfordii and Tripterygium regelii, is a natural compound that has recently received great attention due to its specific structure, promising bioactivities, and excellent safety profile when consumed at low doses [5]. Some studies have identified the positive pharmacological activities of CLT in a variety of cancer types, suggesting CLT as a potential antitumor drug [6, 7]. CLT exerts its antitumor effect in various ways, including increasing reactive oxygen species (ROS) levels and causing ROS-dependent endoplasmic reticulum (ER) stress, mitochondrial dysfunction, and apoptosis by inhibiting peroxiredoxin-2 (PRDX2, an antioxidant enzyme in cancer cells) [8]. Recent studies have demonstrated that CLT has an excellent performance in inhibiting tumor growth [9, 10], including CRC [11]. However, the low solubility and bioavailability of CLT limits its potential for clinical application. At the same time, protective autophagy induced by CLT counteracts its proapoptotic effect [10]. Thus, overcoming these problems can be a new strategy enabling CLT to improve its antitumor effects and promote its clinical application in cancer treatment.

Photothermal therapy (PTT) is an attractive compliment to traditional cancer treatments owing to its unique advantages, such as noninvasiveness, low toxicity, negligible drug resistance, and minimal side effects [12]. PTT can produce high heat to induce apoptosis, which is expected to enhance the effect of chemotherapy. The new indocyanine green (IR820), a new structural analog of indocyanine green (ICG), has been approved by the Food and Drug Administration (FDA) for clinical use, having the merits of strong near-infrared absorbance, excellent biocompatibility, and easy metabolism [13, 14]. However, due to its poor targeting ability, insufficient antitumor efficacy, and short retention time, the clinical application of IR820 has to date been limited [15]. Therefore, PTT including IR820, is not used as a monotherapy but is usually combined with other treatments to promote apoptosis in cancer cells [16–19]. For example, PTT has displayed an obviously synergistic effect with chemotherapy to improve its antitumor effects, especially using nanoplatforms to enhance targeting ability, retention time, and bioavailability [20].

**Scheme 1:**
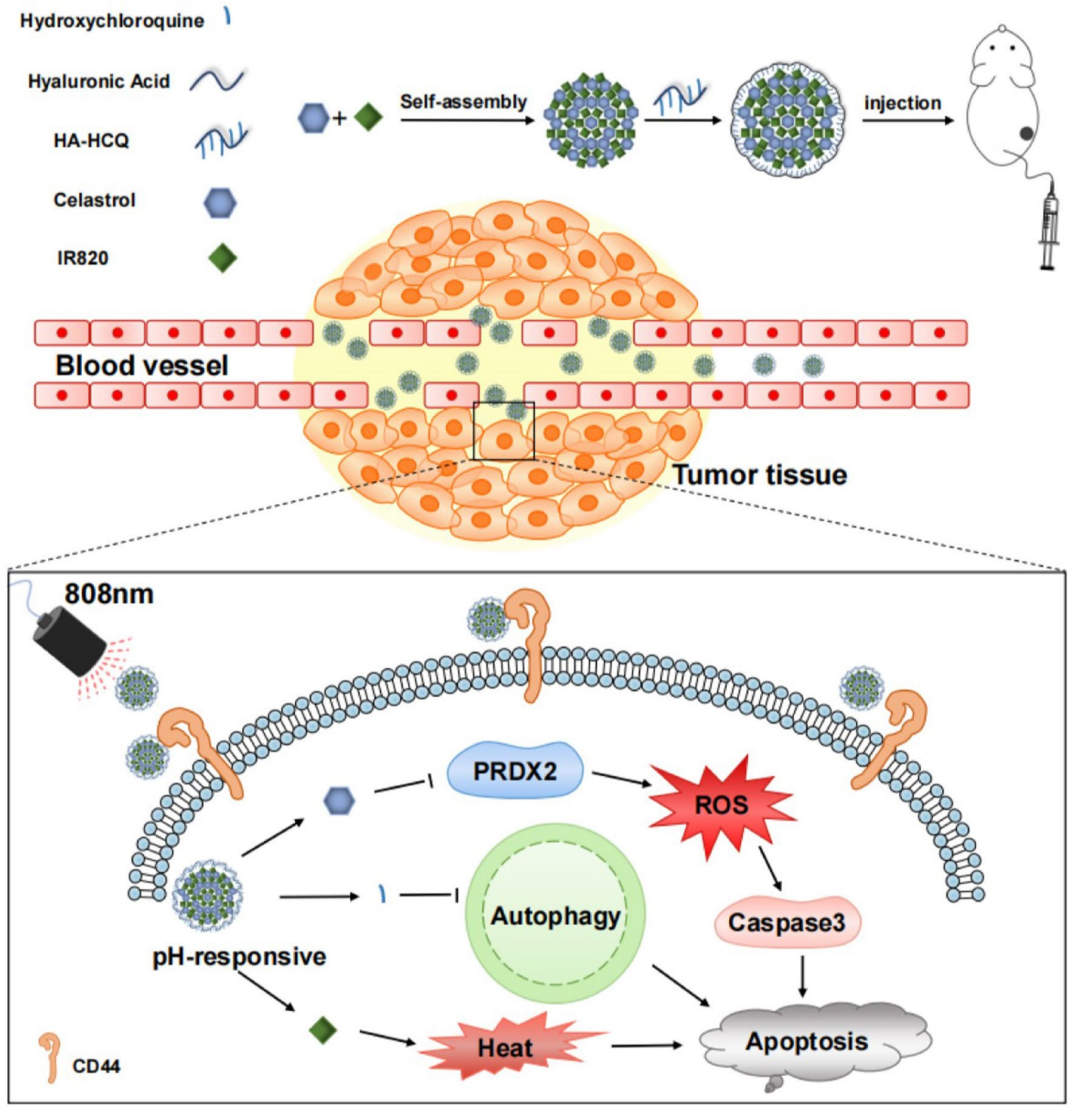
A specific-targeted nanomodulator, capable of modulation of autophagy in the tumor microenvironment, was designed for enhanced colorectal cancer chemo-phototherapy

Herein, we present a chemo-photothermal nanoparticle capable of augmenting apoptosis against CRC growth by co-assembling CLT and IR820 through modification with HA-conjugated hydroxychloroquine (HCQ) (HCR NPs). The HCR NPs not only improved the solubility of CLT but also enhanced the targeting ability, retention time, and bioavailability of CLT and IR820. HCQ, a well-known autophagy inhibitor [21], inhibits autophagy to enhance apoptosis induced by CLT. Hyaluronic acid (HA), which has excellent water solubility, is an excellent carrier in nanomaterials owing to its ability to target CD44 expressed on cancer cells [22, 23]. Therefore, we conjugated HCQ to HA as a new carrier for loading CLT and IR820, to regulate autophagy and improve the water solubility and targeting ability of CLT on the one hand and further enhance apoptosis via the combination with PTT on the other. The HCR NPs show a great therapeutic effect on CRC both in vitro and in vivo by accelerating ROS accumulation through PRDX2 inhibition and amplifying apoptosis by HCQ-mediated autophagy inhibition and IR820-modulated PTT. Thus, HCR NPs provide a new approach for the clinical application of CLT by improving its water solubility and inhibiting protective autophagy through chemo-phototherapy nanoplatforms, which may afford a new means for the treatment of CRC (Scheme 1).

## Materials and methods

### Materials

CLT (MED80120) was purchased from Medbio Pharmaceutical Technology Company. IR-820 (N119962) and hydroxychloroquine sulfate (H141480) were purchased from Shanghai Aladdin Biochemical Co., Ltd. Hyaluronic acid (H909935) was purchased from Shanghai Macklin Biochemical Co., Ltd. Penicillin-streptomycin solution (SV30010) was manufactured by HyClone Laboratories. Fetal bovine serum albumin (FBS) (FSP500) was purchased from ExCell Bio. Dulbecco’s modified Eagle’s medium (DMEM) (C11995500BT) was purchased from ThermoFisher Biochemical Products Co., Ltd. The ROS assay kit (S0033S), LDH assay (C0016), Calcein AM/PI cell viability/cytotoxicity assay kit (C2015S), and mitochondrial membrane potential assay kit with JC-1 (C2006) were provided by Beyotime Biotechnology. An Annexin V-FITC/PI apoptosis detection kit (40302ES20) was purchased from Yeasen Biotech Co., Ltd. EdU assay kit (C10310) was provided by Guangzhou RiboBio Co., Ltd. 3-(4,5-dimethyl-2-thiazoyl)-2,5-diphenyltetra tetrazolium bromide (MTT) (M2128) and N-acetyl cysteine (NAC) (A9165) were obtained from Sigma-Aldrich Co., Ltd. DQ-BSA Red (D-12,051) was purchased from ThermoFisher Scientific Co., Ltd. We thank the Pub-lab of West China School of Basic Medical Sciences & Forensic Medicine, Sichuan University for providing several laboratory instruments.

### Preparation of HA-HCQ

HA-HCQ was synthesized by an esterification reaction in the presence of DCC, and 4-DMAP. Briefly, 800 mg HA, 100 mg DCC and 40 mg 4-DMAP were added to a 30 mL dimethyl sulfoxide (DMSO)/H_2_O (V/V, 1:1) mixture solution and stirred for 1 h at 60 °C to activate the carboxylic group of HA. Then, 86.8 mg HCQ was added to this reaction system and stirred at 300 rpm for an additional 24 h at 60 °C to produce HA-HCQ. After the reaction, the resultant solution was transferred into a dialysis bag (MWCO: 3.5 kDa) to dialyze for 48 h with frequent exchanges of deionized water. The dialyzed solution was centrifuged at 10 000 rpm to remove water-insoluble byproducts, followed by lyophilization [24].

### Preparation of CLT-IR820 and HCR NPs

CLT-IR820 nanoparticles were fabricated with CLT and IR820. Briefly, CLT (2.25 mg) and IR820 (8.50 mg) were dissolved in 1 mL methanol. The above CLT solution (1 mL) and IR820 solution (1 mL) were stirred (1000 rpm) for 20 min at 25 °C. After that, 5 mL distilled water was added to the mixture and stirred for another 10 min. Finally, methanol was removed by rotary evaporation for 20 min at 37 °C.

HCR NPs were prepared in the same manner, except HA-HCQ aqueous solution (5 mg/mL) was used instead of distilled water.

### Characteristics of CLT-IR820 and HCR NPs

A transmission electron microscope (TEM, HT7800, Electron Microscope Laboratory, West China School of Basic Medical Sciences & Forensic Medicine, Sichuan University) and a zeta sizer nano analyzer (Malvern) were used to characterize the morphology and size distribution of the nanoparticles, respectively.

### The release of HCR NPs in vitro

The pH sensitivity of the HCR NPs was determined by the dialysis method to test the in vitro drug release performance. For pH sensitivity-based drug release, 1 mL of the HCR NPs solution (consisting of 450.6 µg CLT) in the dialysis bag (MWCO: 3.5 kDa) was added into phosphate buffer with a volume of 20 mL at pH 7.4 or 5.0. The release media were shaked at 100 rpm and 37.0 °C. A part of the release media (1 mL) was taken out, and simultaneously fresh release media (1 mL) were supplied at different times.

### The photothermal capability of HCR NPs in vitro

The photothermal capability of HCR NPs was measured at 1, 2, 3, 4, and 5 min with various concentrations (0, 20, 40, 80, 160 µΜ) under NIR laser irradiation (808 nm, 1 W/cm^2^, 5 min).

### Uptake of HCR NPs by DLD-1 cells and HCT116 cells

DLD-1 cells and HCT116 cells were seeded on 6-well plates and incubated in complete medium for 24 h at 37 ℃. The medium was then replaced with 1 mL of fresh medium containing HCR NPs (6 µM), and the cells were incubated for another 0 h, 1 h, 2 h, 4 h, and 6 h. After washing with PBS three times, flow cytometry was used to measure the fluorescence intensity.

### Cell viability assay

DLD-1 and HCT116 cells were seeded on 96-well plates (5000 cells/well) and incubated in complete medium for 24 h at 37 ℃. The next day, the cells were treated with HA-HCQ, CLT, IR820, CLT + IR820, CLT-IR820, or HCR NPs at various concentrations for 4 h at 37 ℃. The medium was then replaced with complete medium. After that, the IR820, CLT + IR820, CLT-IR820, and HCR NPs groups were irradiated with or without an 808 nm laser at 1 W/cm^2^ for 40 s. Then, the cells were incubated for 24 h at 37 ℃. After incubation, cell viability was evaluated by using the MTT assay.

### Cell proliferation and cytotoxicity assay

DLD-1 and HCT116 cells were seeded on 96-well plates (5000 cells/well) and incubated in complete medium for 24 h at 37 ℃. The next day, the cells were treated with CLT, CLT + IR820, CLT-IR820, or HCR NPs at various concentrations for 4 h at 37 ℃. Medium was then replaced with complete medium. After that, the group of CLT + IR820, CLT-IR820, and HCR NPs were irradiated with 808 nm laser at 1 W/cm^2^ for 40 s. Then continued to incubate 24 h at 37 ℃. After incubation, cell proliferation was evaluated by EdU assay and cell cytotoxicity was evaluated by using the LDH assay.

The Live and Dead assay was also used to confirm the cytotoxicity of HCR NPs to tumor cells. After being seeded in 6-well plates and incubated for 24 h, DLD-1 cells and HCT116 cells were treated with CLT, CLT + IR820, CLT-IR820, and HCR NPs at a concentration of 6 µM. After 4 h, the medium was replaced with complete medium. The CLT + IR820, CLT-IR820, and HCR NPs groups were irradiated with an 808 nm laser at 1 W/cm^2^ for 2 min. Then, the cells were incubated for 24 h at 37 ℃. After that, the cells were stained with the Calcein/PI cell viability assay kit following the manufacturer’s recommendations.

The long-term effects on tumor cell proliferation were analyzed with a colony formation assay. Cells were seeded in 24-well plates (500 cells/well) and treated with HA-HCQ, CLT, IR820 + Laser, CLT + IR820 + Laser, CLT-IR820 + Laser, and HCR NPs + Laser at a concentration of 5 µM. The medium was changed every 3 d. After 2 weeks, the colonies were stained with crystal violet for 30 min and washed 3 times.

### Apoptosis assay

DLD-1 and HCT116 cells were seeded on 6-well plates and incubated in complete medium for 24 h at 37 ℃. The second day, the cells were treated with 6 µM CLT, CLT + IR820, CLT-IR820, and HCR NPs. After 4 h, the medium was replaced with complete medium. The CLT + IR820, CLT-IR820 and HCR NPs groups were irradiated with an 808 nm laser at 1 W/cm^2^ for 2 min. Then, the cells were incubated for 12 h at 37 ℃. Then, the cells were measured using the Annexin V-FITC/PI apoptosis detection kit, following the manufacturer’s recommendations.

### The detection of intracellular ROS

DLD-1 cells and HCT116 cells were seeded on 6-well plates and incubated in complete medium for 24 h at 37 ℃, then treated with 6 µM CLT, CLT + IR820, CLT-IR820, and HCR NPs. After 4 h, the medium was replaced with complete medium. The CLT + IR820, CLT-IR820 and HCR NPs groups were irradiated with an 808 nm laser at 1 W/cm^2^ for 2 min. Then, the cells were incubated for 12 h at 37 ℃. Then, intracellular ROS levels were measured using the ROS assay kit following the manufacturer’s recommendations.

### In vivo biodistribution of HCR NPs

All in vivo assays were in accordance with the animal protection guidelines of Southwest Medical University. BALB/c nude male mice (5–6 weeks old) were purchased from Chengdu Yaokang Bioscience Co., Ltd. (Beijing, China). DLD-1 cells (1.0 × 10^7^ cells) were subcutaneously injected into the right hindlimb region to establish CRC-bearing mouse models. The CRC-bearing mice were randomly divided into the following three groups: (1) IR820, (2) CLT-IR820, and (3) HCR NPs. When the tumor size of the mice grew to approximately 100 mm^3^, mice received free IR820, CLT-IR820 or HCR NPs (4 mg/kg of an equivalent amount of IR820) via tail vein injection. At 2, 4, 6, 8, 12, and 24 h after administration, mice were anesthetized and imaged using an IVIS Lumina III (CLS136334, PerkinElmer). The mice were sacrificed 24 h post-administration, and the tumors and major organs were harvested and subjected to ex vivo fluorescence imaging as mentioned above. The fluorescence from each organ was analyzed by the instrument software [25].

### Animal models

All in vivo assays were in accordance with the animal protection guidelines of Southwest Medical University. BALB/c nude male mice (5–6 weeks old) were purchased from Chengdu Yaokang Bioscience Co., Ltd. (Beijing, China). DLD-1 cells (1.0 × 10^7^ cells) were subcutaneously injected into the right hindlimb region to establish CRC-bearing mouse models. The CRC-bearing mice were randomly divided into the following three groups (n = 5/group): (1) saline, (2) CLT-IR820 with laser, and (3) HCR NPs with laser. When the tumor size of the mice grew to approximately 100 mm^3^ (n = 5/group), mice in the treatment groups received CLT-IR820 or HCR NPs (4 mg/kg of an equivalent amount of IR820) via tail vein injection every two days, whereas the control mice received saline only every two days. At the same time, tumor volume and body weight were recorded. The tumors of the CLT-IR820 and HCR NPs groups were irradiated with an 808 nm laser at 1 W/cm^2^ for 5 min at 4 h after intravenous injection.

### Statistical analysis

All statistical analysis and graphics were performed using GraphPad 8 software (GraphPad, La Jolla, CA, USA). A one-way ANOVA or Student’s t-test was used to analyze statistical differences. All data are presented as the mean with SD from at least three individual experiments. A value of *P* < 0.05 was considered statistically significant.

## Results and discussion

### Synthesis and characterization of HCR NPs

**Fig. 1:**
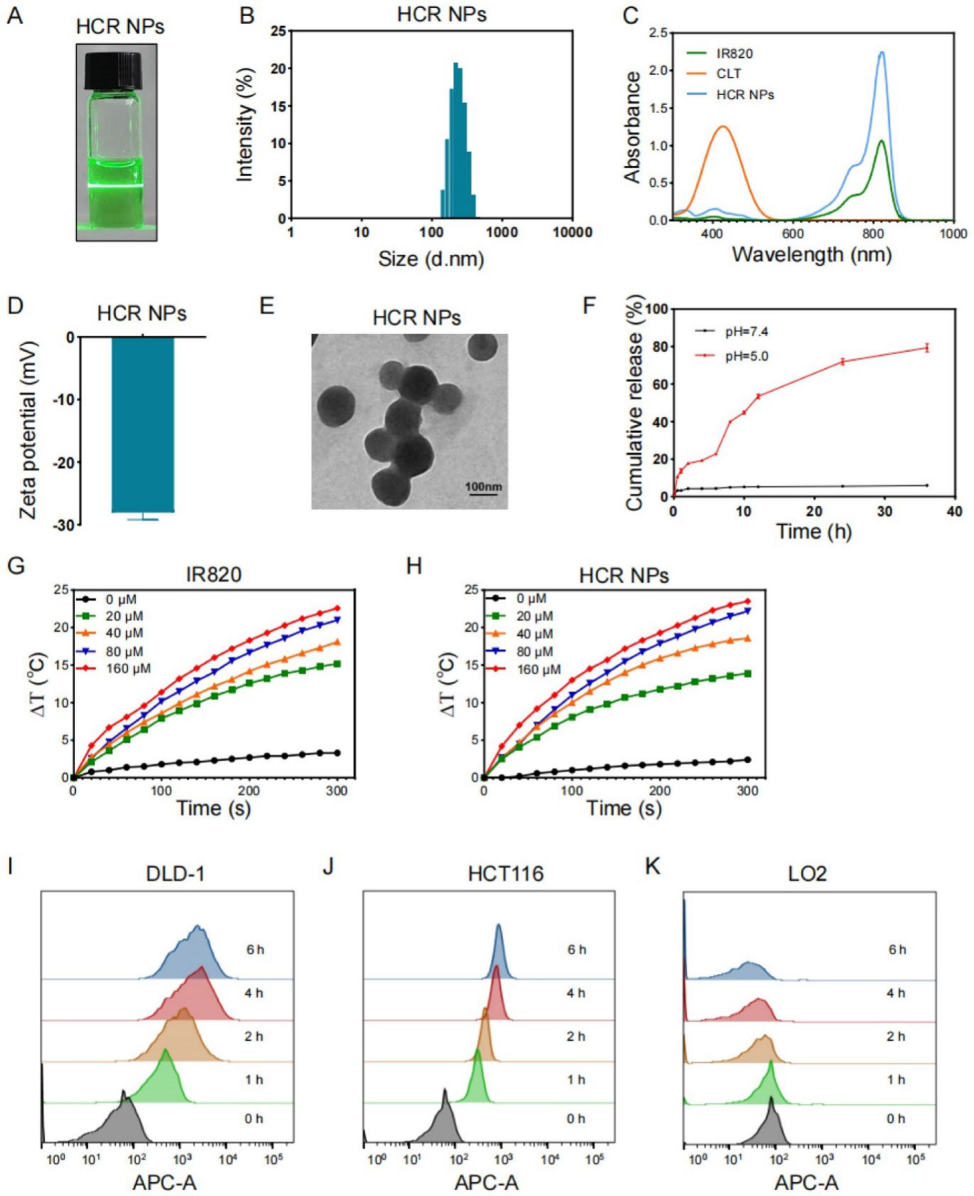
Synthesis and Characterization of HCR NPs and in vitro cellular uptake. **A** The Tyndall effect of HCR NPs. **B** Size distribution of HCR NPs. **C** UV–vis absorption spectra of HCR NPs. **D** Zeta potential of HCR NPs. **E** TEM image of HCR NPs. Scale bar: 100 nm. **F** The release of CLT at pH 5.0 and 7.4. **G**, **H** The photothermal activity of IR820 and HCR NPs dispersed in water at various concentrations (λ = 808 nm, P = 1 W/cm^2^; 5 min). **I**-**K** Flow cytometric results of the cellular uptake of HCR NPs in DLD-1, HCT116, and LO2 cells after incubation for the indicated times

HA-HCQ was obtained by conjugating HCQ to HA through amide bonds (Additional file 1: Fig. S1A). ^1^ H NMR results showed that HA-HCQ was successfully synthesized (Additional file 1: Fig. S1B). CLT-IR820 and HCR NPs were successfully prepared following a three-step approach, as outlined in Fig. 1 and Additional file 1: Fig. S1. The Tyndall effect of CLT-IR820 and HCR NPs was clearly visible (Additional file 1: Fig. S1D and Fig. 1A) even after 14 days (Additional file 1: Fig. S1C), suggesting excellent stability of the HCR NPs. Dynamic light scattering (DLS) results showed that the diameters of CLT-IR820 and HCR NPs were 101.3 nm (Additional file 1: Fig. S1E) and 251.9 nm (Fig. 1B), respectively, which were suitable for passive tumor targeting due to the enhanced permeability and retention (EPR) effect. The increased diameter of the HCR NPs suggested that HA-HCQ was successfully modified. Additionally, UV-vis absorbance spectra of CLT-IR820 and IR820 were measured to confirm successful synthesis (Additional file 1: Fig. S1F and Fig. 1C). Moreover, the zeta potential of CLT-IR820 (-31.9 mV (Additional file 1: Fig. S1G)) and HCR NPs (-28.1 mV (Fig. 1D)) was stable, guaranteeing a prolonged blood circulation time. Transmission electron micrographs (TEM) confirmed that CLT-IR820 and HCR NPs had a well-defined spherical nanostructure (Additional file 1: Fig. S1H and Fig. 1E). Moreover, the loading efficacy of IR820 and CLT was about 23.8% and 6.3%, respectively. In addition, only about 5.96% CLT was released from HCR NPs in pH 7.4 PBS, whereas 79.42% CLT was released in pH 5.0 PBS at 36 h. Therefore, our HCR NPs could have a selective release in tumor sites due to its acidic microenvironment (Fig. 1F).

The photothermal capacity of the HCR NPs was then detected by measuring the temperature change during laser irradiation (808 nm, 1 W/cm^2^) in vitro. The temperature of both free IR820 and HCR NPs was increased in a concentration- and time-dependent manner (Fig. 1G-H). Thus, the synthesis of HCR NPs did not influence the heating capacity of IR820, and HCR NPs could be used as efficient nanoparticles for PTT.

Intracellular uptake of nanoparticles is critical for efficient drug delivery. Hence, we chose two CRC cell lines (HCT116 and DLD-1) to assess the effectiveness of cell uptake. Flow cytometry results indicated that HCR NPs gradually accumulated over time and reached maximum levels after 4 h (Fig. 1I-J). At the same time, we also assessed the effectiveness of cell uptake in LO2 (a human normal liver cell) and the result showed that HCR NPs cannot be absorbed (Fig. 1 K), indicating that HCR NPs can effectively accumulate in tumor cells by CD44 receptors-mediated active targeting.

### Antitumor effect of HCR NPs against CRC

**Fig. 2:**
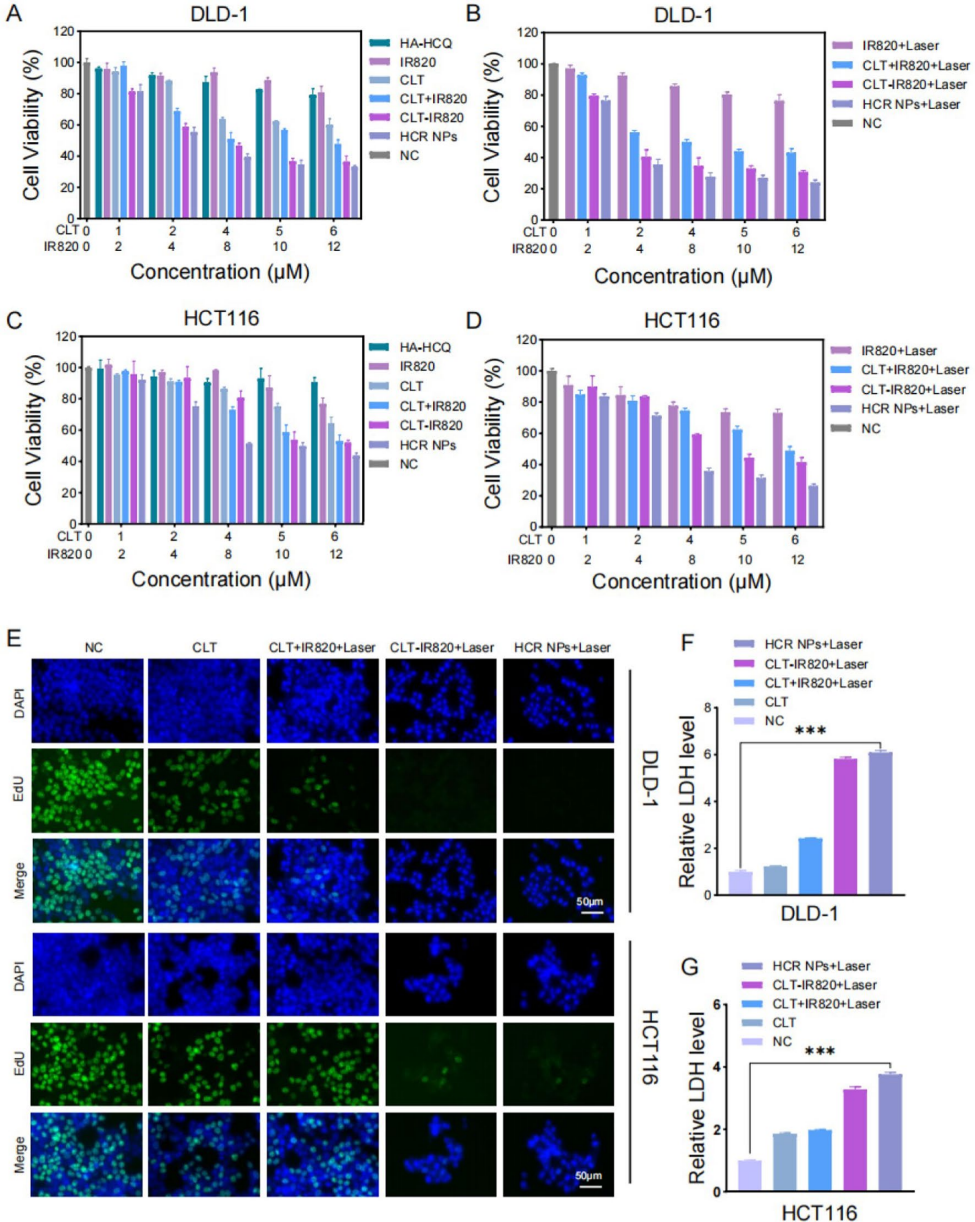
In vitro anti-colorectal cancer effect of HCR NPs. **A**–**D** The viability of DLD-1 and HCT116 cells following treatment with HA-HCQ, IR820, CLT, CLT + IR820, CLT-IR820, HCR NPs, IR820 + Laser, CLT + IR820 + Laser, CLT-IR820 + Laser, and HCR NPs + Laser (λ = 808 nm, P = 1 W/cm^2^; 40 s) at different concentrations. **E** The proliferation of DLD-1 and HCT116 cells treated with CLT, CLT + IR820 + Laser, CLT-IR820 + Laser, and HCR NPs + Laser measured by the EdU assay. Scale bar: 50 μm. (λ = 808 nm, P = 1 W/cm^2^; 40 s). **F**, **G** LDH assay of DLD-1 and HCT116 cells cocultured with CLT, CLT + IR820 + Laser, CLT-IR820 + Laser, and HCR NPs + Laser. (λ = 808 nm, P = 1 W/cm^2^; 40 s). ***P < 0.001

The anti-CRC properties of HCR NPs were first examined using a 3-(4,5-dimethylthiazol-2-yl)-2,5-diphenyltetrazolium bromide (MTT) assay in different treatment groups, including HA-HCQ, CLT, IR820, CLT + IR820, CLT-IR820, and HCR NPs. In another group IR820, CLT + IR820, CLT-IR820, and HCR NPs were irradiated with an 808 nm laser at 1 W/cm^2^ for 40 s. All treatments inhibited CRC cell viability and showed concentration-dependent effects. The group using HCR NPs with laser treatment had the best inhibitory effect on cell viability, indicating the realization of specific-targeted chemo-phototherapy (Fig. 2A-D). In addition, the results of the EdU assay (Fig. 2E and Additional file 1: Fig. S2A, B), LDH assay (Fig. 2F-G), colony formation assay (Additional file 1: Fig. S2C–E), and Calcein AM/PI cell viability assay (Additional file 1: Fig. S2F) were in agreement with those of the MTT assay. In summary, these results indicated that the HCR NPs have excellent anti-colorectal cancer capability by combining chemo-photothermal therapy in vitro.

### HCR NPs induce apoptosis in CRC cells

**Fig. 3:**
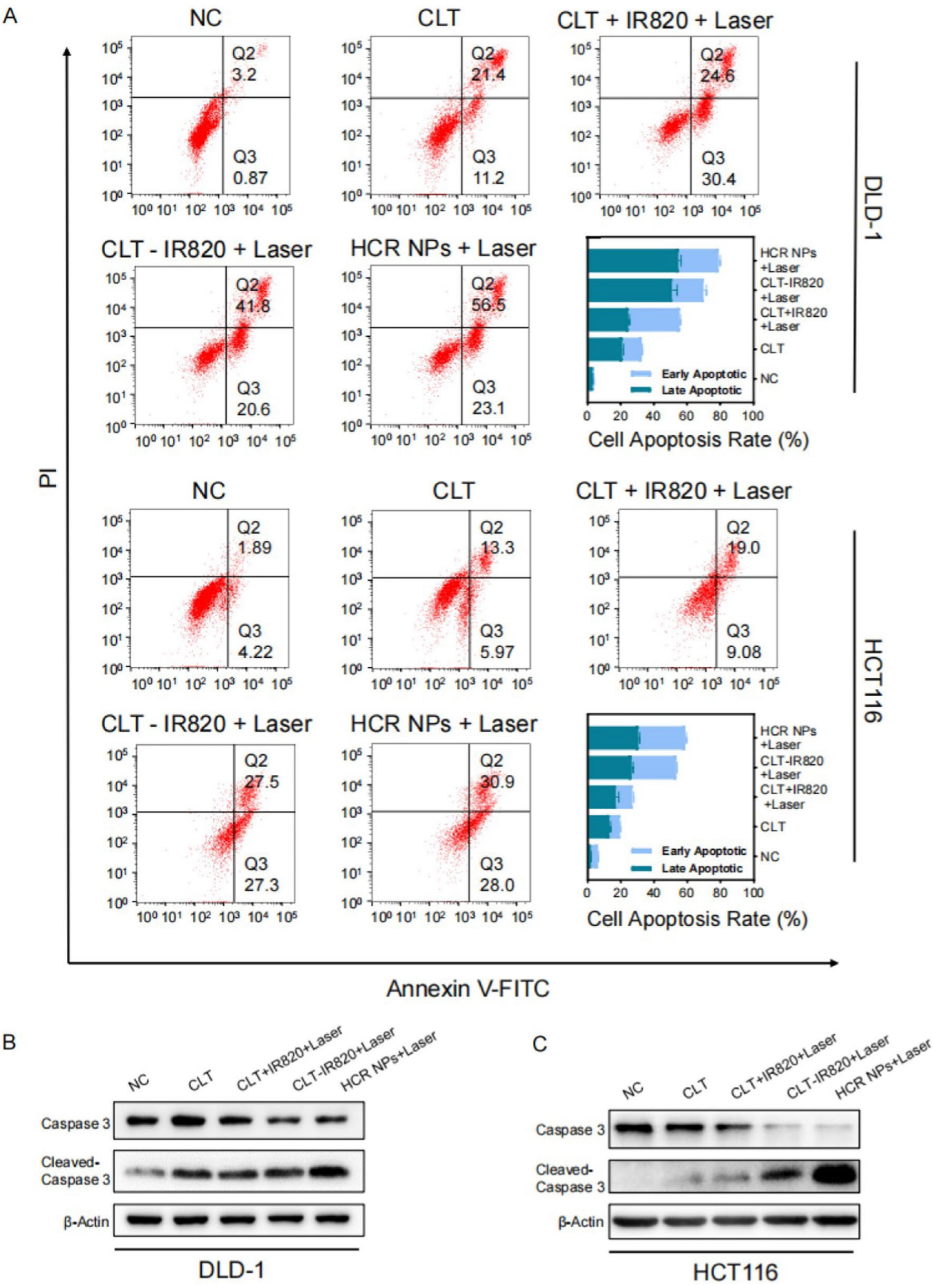
HCR NPs induce apoptosis in colorectal cancer cells. **A** Apoptosis of CLT, CLT + IR820 + Laser, CLT-IR820 + Laser, and HCR NPs + Laser was evaluated by Annexin V-FITC/PI staining in DLD-1 and HCT116 cells. Quantification of the apoptotic cell ratio is shown. (λ = 808 nm, P = 1 W/cm^2^; 2 min). **B**, **C** Immunoblot analysis of apoptotic markers in DLD-1 and HCT116 cells treated with CLT, CLT + IR820 + Laser, CLT-IR820 + Laser, or HCR NPs + Laser. (λ = 808 nm, P = 1 W/cm^2^; 2 min)

An Annexin V/propidium iodide (PI) double staining assay was used to detect the apoptosis of CRC cells [26]. The flow cytometry results illustrated that HCR NPs showed a significant pro-apoptotic effect on CRC cells compared to other groups (Fig. 3A). Consistently, the expression of apoptotic markers, including caspase3, and cleaved-caspase3, was changed after HCR NPs treatment (Fig. 3B, C). At the same time, the intracellular mitochondrial membrane potential was markedly decreased upon HCR NPs treatment, as monitored by JC-1 staining, indicating that HCR NPs could cause early apoptosis (Additional file 1: Fig. S3A). Together, these results indicate that HCR NPs can stimulate apoptosis in CRC cells. CLT has been reported to induce apoptosis and protective autophagy, which counteract each other [10]. Therefore, we conjugated HCQ to HA to prevent the inhibition of autophagy on CLT-induced apoptosis. DLD-1 cells and HCT116 cells incubated with BODIPY-conjugated bovine serum (DQ-BSA, red) were treated with HA-CLT-IR820 + Laser or HCR NPs + Laser, and the fluorescence of DQ-BSA was decreased due to blocked proteolytic degradation in HCR NPs-treated cells which indicated that autophagy was inhibited (Additional file 1: Fig. S3B) [27]. Moreover, the upregulated expression of microtubule-associated protein light chain 3 (LC3)-II and p62 indicated that autophagy could be inhibited by HCQ (Additional file 1: Fig. S3C).

### The cytotoxicity of HCR NPs is dependent on ROS

**Fig. 4:**
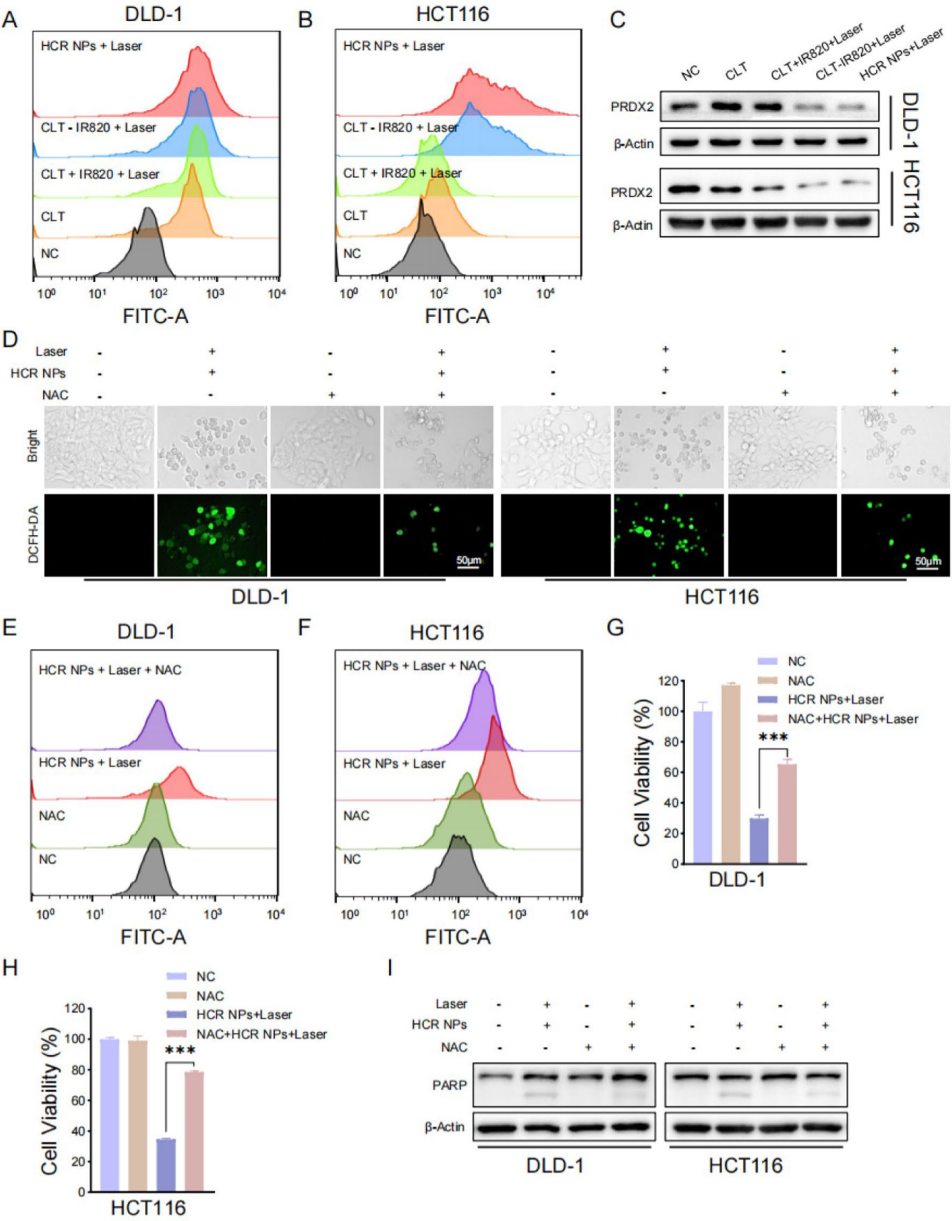
HCR NPs-mediated ROS production promotes colorectal cancer cell death. **A**, **B** Flow cytometry analysis of intracellular ROS generation in DLD-1 and HCT116 cells treated with CLT, CLT + IR820 + Laser, CLT-IR820 + Laser, or HCR NPs + Laser using DCFH-DA as a probe. (λ = 808 nm, P = 1 W/cm^2^; 2 min). **C** Immunoblot analysis of PRDX2 in DLD-1 and HCT116 cells treated with CLT, CLT + IR820 + Laser, CLT-IR820 + Laser, and HCR NPs + Laser. (λ = 808 nm, P = 1.0 W/cm^2^; 2 min). **D** Fluorescent images of DCFH-DA-stained DLD-1 cells and HCT116 cells treated with HCR NPs + Laser in combination with or without NAC treatment. Scale bar: 50 μm. (λ = 808 nm, P = 1.0 W/cm^2^; 2 min). **E**, **F** Flow cytometry analysis of intracellular ROS generation in HCR NPs + Laser -treated DLD-1 and HCT116 cells with or without NAC treatment using DCFH-DA as a probe. (λ = 808 nm, P = 1 W/cm^2^; 2 min). **G**, **H** Cell viability of DLD-1 cells and HCT116 cells treated with HCR NPs + Laser in combination with or without NAC treatment. (λ = 808 nm, P = 1.0 W/cm^2^; 40 s). ***P < 0.001. **I** Immunoblot analysis of apoptotic marker in DLD-1 and HCT116 cells treated with HCR NPs + Laser in combination with or without NAC treatment. (λ = 808 nm, P = 1.0 W/cm^2^; 2 min). ***P < 0.001

Abundant evidence indicates that intracellular ROS accumulation may induce apoptosis in cancer cells [28–30]. CLT can directly target PRDX2 to inhibit its activity, which leads to increased intracellular ROS levels and subsequent apoptosis [8]. Therefore, the 2’,7’-dichlorofluorescein diacetate (DCFH-DA) probe was used to assess the intracellular level of ROS after different treatments [31]. The flow cytometry results showed that HCR NPs induced a massive accumulation of cellular ROS in CRC cells (Fig. 4A, B). Moreover, the expression of PRDX2 in the HCR NPs group was significantly inhibited (Fig. 4 C). N-acetyl cysteine (NAC), an ROS scavenger, was used to restore ROS levels. The ROS level of the HCR NPs group was obviously decreased with NAC treatment (Fig. 4D–F and Additional file 1: Fig. S4A, B). In addition, the high ROS level contributed to increased cytotoxicity in CRC cells. The cell viability of the HCR NPs-treated group could be rescued by treatment with NAC (Fig. 4G, H). The results of the EdU assay were in agreement with those of the MTT assay (Additional file 1: Fig. S4C–E). Consistently, the expression of apoptotic marker was also recovered with NAC treatment (Fig. 4I). In addition, the group of IR820 + Laser did not produce significant amounts of ROS, indicating its limit potential for photodynamic therapy (Additional file 1: Fig. S4F, G). The accumulation of intracellular ROS is mainly due to the inhibition of PRDX2 by CLT but not PTT. Collectively, these data suggested that HCR NPs promote ROS accumulation by decreasing PRDX2 expression, thus displaying cytotoxicity in CRC cells.

### The photothermal capacity and anti-CRC effect of HCR NPs in vivo

**Fig. 5:**
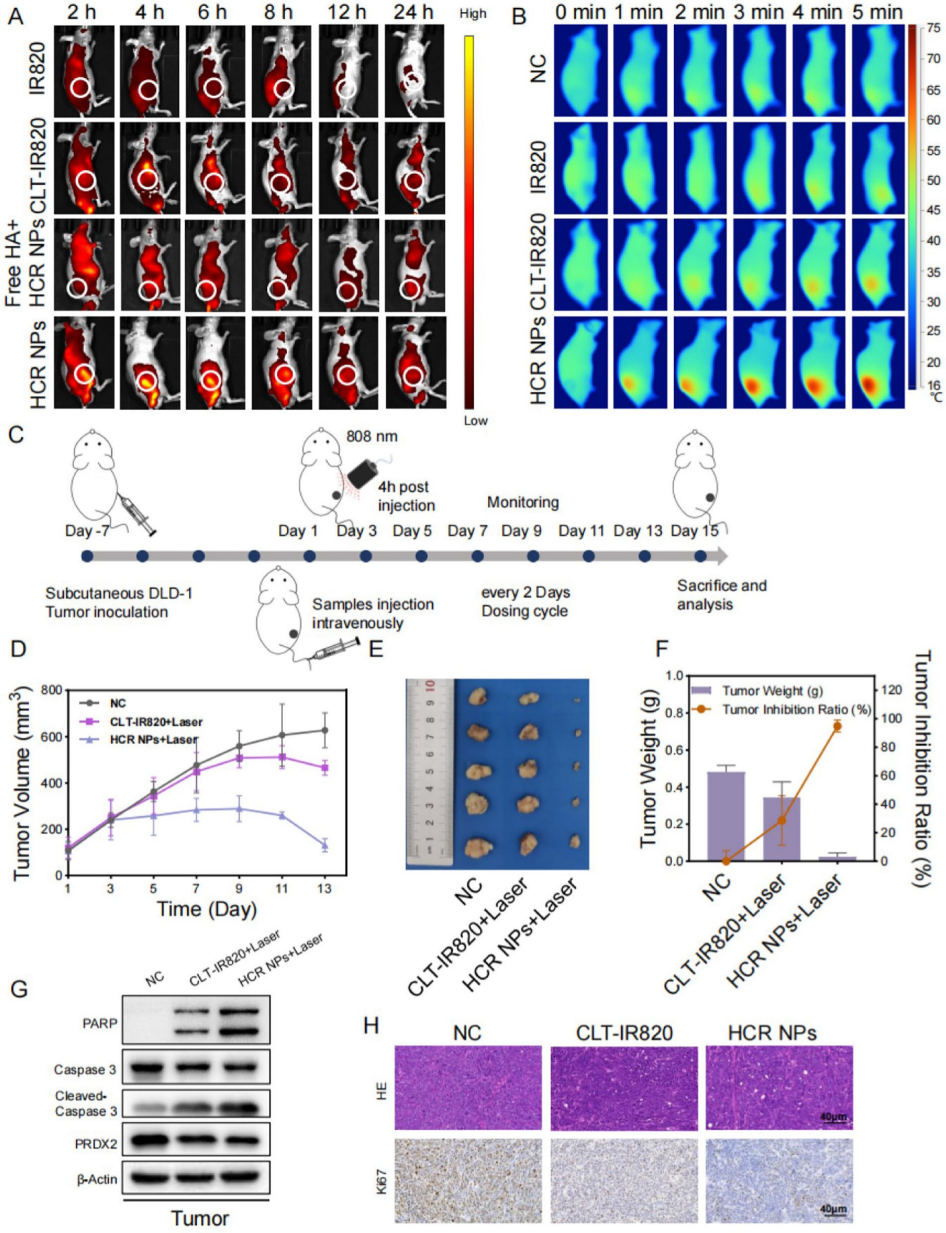
The photothermal capacity and anti-CRC effect of HCR NPs in vivo. **A** In vivo distribution of IR820, CLT-IR820, free HA + HCR NPs, and HCR NPs at various times. **B** In vivo thermal imaging of different groups of treated mice. (λ = 808 nm, P = 1 W/cm^2^; 5 min). **C** Schematic illustration of the tumor treatment procedure in vivo. **D** The volume of tumors from each group (5 mice per group) was measured at the indicated time points. **E** Representative images of isolated tumors. **F** The weight of individual tumors and the inhibition ratio. **G** Immunoblot analysis of apoptotic markers and PRDX2 in tumor tissues treated with vehicle, CLT-IR820 or HCR NPs intravenously through the tail vein at the end of the treatment period. (λ = 808 nm, P = 1 W/cm^2^; 5 min). **H** Representative H&E staining and Ki67 immunohistochemistry images at the end of the treatment period of mice treated with vehicle, CLT-IR820 or HCR NPs intravenously through the tail vein. Scale bar: 40 μm. (λ = 808 nm, P = 1 W/cm^2^; 5 min)

Mice bearing DLD-1 tumors were injected with normal saline, IR820, CLT-IR820, or HCR NPs, as described in Additional file 1: Fig. S5A. To further detect the biodistribution of HCR NPs, we monitored the fluorescence signal of HCR NPs at various time points using a living imaging system (Fig. 5A). The results indicated that HCR NPs had a maximum fluorescence intensity at the tumor site 4 h after intravenous injection. Thus, we chose laser irradiation 4 h after intravenous administration for subsequent PTT. Moreover, free IR820, CLT-IR820 and free HA + HCR NPs (HCR NPs with pre-injection of free HA) were not significantly enriched at the tumor site, showing that HCR NPs had a significant targeting effect. Furthermore, the attenuation of the fluorescent intensity of free IR820 was much faster than that of HCR NPs, with the fluorescent signal of HCR NPs lasting for 24 h. In order to further confirm the role of HA in the active targeting of HCR NPs to CD44 receptors of tumor, a high dose of HA polymer was intravenously injected prior to the administration of HCR NPs to saturate CD44 receptors on tumor cells and the result showed that the group of free HA + HCR NPs (HCR NPs with pre-injection of free HA) had no significant enrichment at the tumor site, supporting that HA enabled HCR NPs with the active tumor targeting ability. The major organs and the tumors were then excised to explore the accumulation efficiency after 24 h. There was a prolonged retention of HCR NPs in the tumor site compared to other groups, indicating that the targeting effect of HA and the EPR effect may promote the accumulation of HCR NPs (Additional file 1: Fig. S5B). As HCR NPs had a maximum fluorescent intensity at the tumor site 4 h after intravenous injection, the tumors were irradiated with an 808 nm laser (1 W/cm^2^) for 5 min at 4 h after injection, and the temperature was monitored by an infrared thermal imager at various time points (Fig. 5B). The results clearly showed that HCR NPs displayed an obvious temperature rise as the time of irradiation was prolonged, increasing to 30.7 ℃ at 5 min (Additional file 1: Fig. S5C). This may profit from the targeting effect of HA and the EPR effect.

Following the in vitro study, the in vivo anti-CRC effect of HCR NPs was evaluated in mice bearing DLD-1 tumors. Mice bearing DLD-1 tumors were stochastically divided into three groups and injected with normal saline, CLT-IR820, and HCR NPs, as illustrated in Fig. 5C. HCR NPs exhibited excellent therapeutic effects based on the visual observation of tumor tissues, monitoring the tumor volume and weight, and calculating the tumor inhibition ratio. The tumor inhibition ratio of the HCR NPs group was close to 95% (Fig. 5D-F). Furthermore, the expression of apoptotic markers, including caspase3, cleaved-caspase3, and PARP, was modulated, and the expression of PRDX2 was also decreased in tumor tissues treated with HCR NPs (Fig. 5G). In addition, HCR NPs reduced cell proliferation, as evidenced by weaker IHC staining of Ki67 in tumor tissues (Fig. 5H). Significantly, the HCR NPs-treated group showed the strongest antitumor efficacy due to the combination of chemotherapy and PTT.

### Evaluation of the biosafety of HCR NPs in vivo

**Fig. 6:**
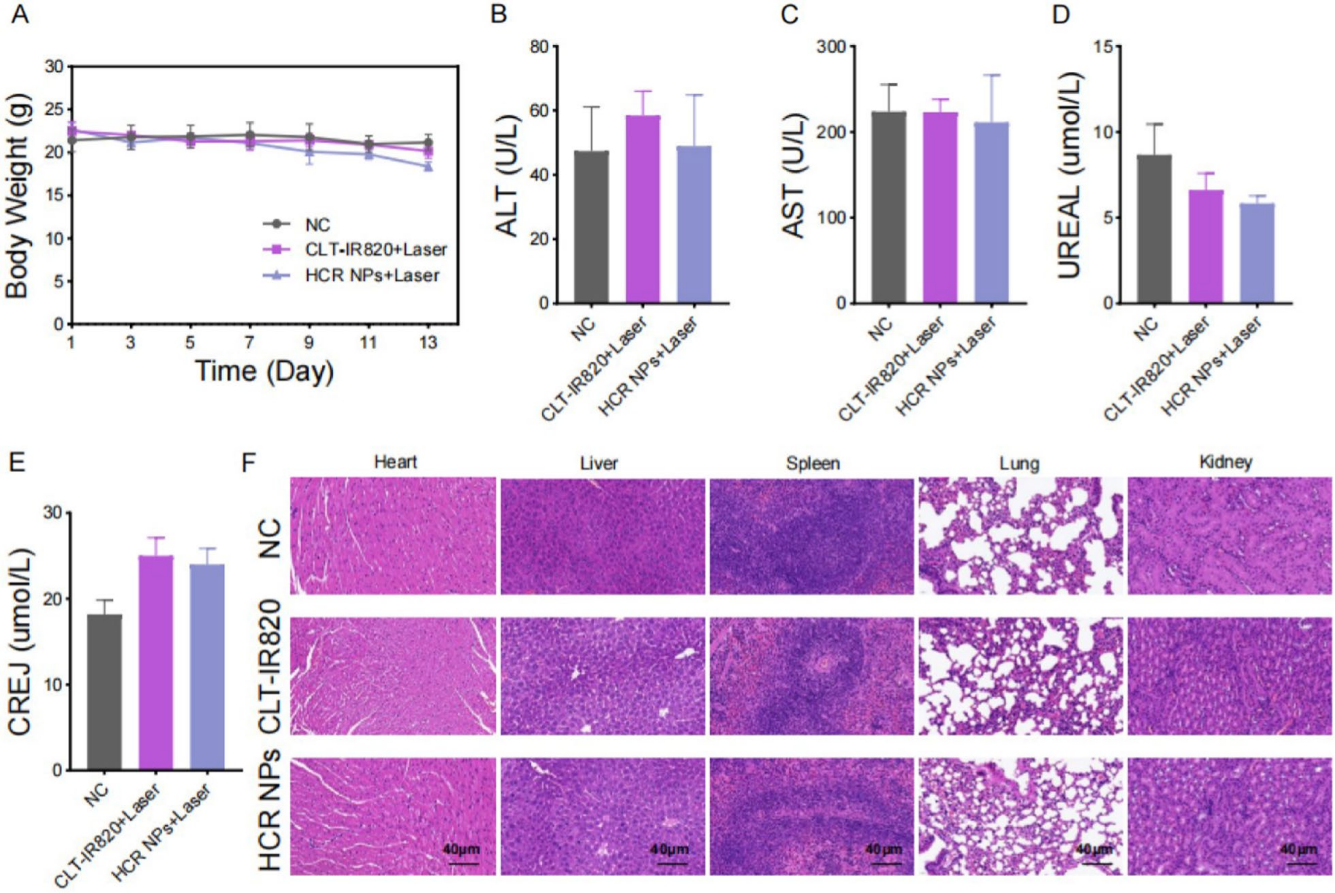
Evaluation of the biosafety of HCR NPs in vivo. **A** The body weight of mice in each group was measured at the indicated time points. **B**-**E** Blood biochemical markers: ALT (**B**); AST (**C**); UREAL (**D**); CREJ (**E**). Each group was tested five times in parallel. **F** Representative heart, liver, spleen, lung, and kidney H&E staining at the end of the treatment period of mice bearing DLD-1 xenografts treated with vehicle, CLT-IR820 or HCR NPs intravenously through the tail vein. Scale bar: 40 μm

No significant weight changes were observed in the mice with different treatments, suggesting good biosafety of HCR NPs (Fig. 6A). Serum biochemical analyses, including alanine aminotransferase (ALT), aspartate aminotransferase (AST), blood urea nitrogen (UREAL), and creatinine (CREJ), are commonly used to reflect the function of the liver and kidney [32, 33]. Serum biochemical analysis of these markers showed no significant fluctuation among NC, CLT-IR820, and HCR NPs (Fig. 6B–E). H&E staining showed no obvious pathological damage to the major organs with HCR NPs treatment (Fig. 6F) [34]. These results all suggested that the HCR NPs had good biosafety in vivo.

## Conclusion

In this study, we successfully synthesized a novel chemo-photothermal nanoparticle, HCR NPs. HCR NPs not only solved the poor solubility and bioavailability of CLT but also improved targeting, retention time, and cellular uptake of drugs. HCR NPs could target cancer cells through HA-mediated enhanced accumulation of drugs at the tumor site. In addition, CLT in HCR NPs induced ROS-mediated apoptosis by inhibiting PRDX2, and IR820 increased the temperature at the tumor site with 808 nm laser irradiation to further enhance the therapeutic effect both in vitro and in vivo. Moreover, HCR NPs displayed good biosafety, with no significant damage to major organs, demonstrating their potential application in the treatment of CRC. Together, our findings should facilitate the development of reasonable and effective strategies for the clinical treatment of CRC.

## Supplementary Information

**Additional file 1: Figure S1.** Preparation and Characterization of CLT-IR820 and stability of HCR NPs. (A) Schematic illustration of the synthesis of HA-HCQ. (B) ^1^H NMR results of HA-HCQ. (C) The Tyndall effect of HCR NPs at the indicated time points. (D) The Tyndall effect of CLT-IR820. (E) Size distribution of CLT-IR820. (F) UV-vis absorption spectra of CLT-IR820. (G) Zeta potential of CLT-IR820. (H) TEM image of CLT-IR820. Scale bar: 100 nm.** Figure S2.** Cytotoxicity of HCR NPs in vitro. (A-B) Quantification of DLD-1 and HCT116 cells co-cultured with CLT, CLT+IR820+Laser, CLT-IR820+Laser, and HCR NPs+Laser in the EDU assay. (λ=808 nm, P =1.0 W/cm^2^; 40 s). ***P < 0.001. (C-E) Representative images of the colony formation of DLD-1 and HCT116 cells treated with HA-HCQ, CLT, IR820+Laser, CLT+ IR820+Laser, CLT- IR820+Laser, and HCR NPs+Laser. (λ=808 nm, P =1 W/cm^2^; 60 s). ***P < 0.001. (F) Cell imaging showing the survival of DLD-1 and HCT116 cells treated with CLT, CLT+ IR820+Laser, CLT- IR820+Laser, and HCR NPs+Laser. Live cells were marked with Calcein-AM (green fluorescence), while dead cells were marked with propidium iodide (red fluorescence). Scale bar: 50 µm. (λ=808 nm, P =1 W/cm^2^; 2 min).** Figure S3.** Apoptosis and inhibition of autophagy. (A) Fluorescent images of JC-1-stained DLD-1 and HCT116 cells, including CLT, CLT+IR820+Laser, CLT-IR820+Laser, and HCR NPs+Laser treatments. Scale bar: 50 μm. (λ=808 nm, P=1 W/cm^2^; 2 min). (B) Fluorescent images of DQBSA-stained DLD-1 and HCT116 cells, including HA-CLT-IR820, and HCR NPs treatments. Scale bar: 50 μm. (λ=808 nm, P=1 W/cm^2^; 2 min). (C) Immunoblot analysis of p62 and LC3-II in DLD-1 and HCT116 cells treated with CLT, HA-CLT-IR820, and HCR NPs. (λ=808 nm, P=1 W/cm^2^; 2 min).** Figure S4.** HCR NPs-mediated ROS inhibited colorectal cancer cell survival. (A-B) Flow cytometry statistical analysis of intracellular ROS generation in DLD-1 and HCT116 cells treated with HCR NPs+Laser with or without NAC treatment using DCFH-DA as a probe. (λ=808 nm, P=1 W/cm^2^; 2 min). (C-E) The proliferation of DLD-1 and HCT116 cells treated with HCR NPs+Laser with or without NAC treatment as measured by EdU assay. Scale bar: 50 μm. (λ=808 nm, P =1 W/cm^2^; 40 s). ***P < 0.001. (F-G) Flow cytometry analysis of intracellular ROS generation in DLD-1 and HCT116 cells treated with IR820+Laser or HCR NPs+Laser using DCFH-DA as a probe. (λ=808 nm, P=1 W/cm^2^; 2 min).** Figure S5.** The photothermal capacity of HCR NPs in vivo. (A) Schematic illustration of the photothermal capacity of HCR NPs in vivo. (B) Fluorescence images of tumors and major organs at 24 h after injection with IR820, CLT-IR820, free HA+HCR NPs, and HCR NPs. (C) In vivo thermal analysis of different groups of treated mice. (λ=808 nm, P=1 W/cm^2^; 5 min).
