# Supplementary material for: Site-specific nanomodulator capable of modulation apoptosis for enhanced colorectal cancer chemo-photothermal therapy
Source: J Nanobiotechnology. 2023 Jan 20;21:24. doi: 10.1186/s12951-023-01779-5 (PMC9863191; doi:10.1186/s12951-023-01779-5)
Supplement: Supplementary file 1 — Additional file 1: Figure S1. Preparation and Characterization of CLT-IR820 and stability of HCR NPs. (A) Schematic illustration of the synthesis of HA-HCQ. (B) 1H NMR results of HA-HCQ. (C) The Tyndall effect of HCR NPs at the indicated time points. (D) The Tyndall effect of CLT-IR820. (E) Size distribution of CLT-IR820. (F) UV-vis absorption spectra of CLT-IR820. (G) Zeta potential of CLT-IR820. (H) TEM image of CLT-IR820. Scale bar: 100 nm. Figure S2. Cytotoxicity of HCR NPs in vitro. (A-B) Quantification of DLD-1 and HCT116 cells co-cultured with CLT, CLT+IR820+Laser, CLT-IR820+Laser, and HCR NPs+Laser in the EDU assay. (λ=808 nm, P =1.0 W/cm2; 40 s). ***P < 0.001. (C-E) Representative images of the colony formation of DLD-1 and HCT116 cells treated with HA-HCQ, CLT, IR820+Laser, CLT+ IR820+Laser, CLT- IR820+Laser, and HCR NPs+Laser. (λ=808 nm, P =1 W/cm2; 60 s). ***P < 0.001. (F) Cell imaging showing the survival of DLD-1 and HCT116 cells treated with CLT, CLT+ IR820+Laser, CLT- IR820+Laser, and HCR NPs+Laser. Live cells were marked with Calcein-AM (green fluorescence), while dead cells were marked with propidium iodide (red fluorescence). Scale bar: 50 µm. (λ=808 nm, P =1 W/cm2; 2 min). Figure S3. Apoptosis and inhibition of autophagy. (A) Fluorescent images of JC-1-stained DLD-1 and HCT116 cells, including CLT, CLT+IR820+Laser, CLT-IR820+Laser, and HCR NPs+Laser treatments. Scale bar: 50 μm. (λ=808 nm, P=1 W/cm2; 2 min). (B) Fluorescent images of DQBSA-stained DLD-1 and HCT116 cells, including HA-CLT-IR820, and HCR NPs treatments. Scale bar: 50 μm. (λ=808 nm, P=1 W/cm2; 2 min). (C) Immunoblot analysis of p62 and LC3-II in DLD-1 and HCT116 cells treated with CLT, HA-CLT-IR820, and HCR NPs. (λ=808 nm, P=1 W/cm2; 2 min). Figure S4. HCR NPs-mediated ROS inhibited colorectal cancer cell survival. (A-B) Flow cytometry statistical analysis of intracellular ROS generation in DLD-1 and HCT116 cells treated with HCR NPs+Laser with or without NAC treatment using DCFH-DA [file 12951_2023_1779_MOESM1_ESM.docx]

**Supplementary materials**

**Site-specific nanomodulator capable of modulation apoptosis for enhanced colorectal cancer chemo-photothermal therapy**

Shuqi Wang^a1^, Li Zhou^e1^, Hailong Tian^b1^, Bowen Li^b^, Miao Su^b^, Qiong Li^b^, Edouard C. Nice^c^, Canhua Huang^b^, Jichun Shao^d*^, Tao He^a*^

*^a^Institute for Cancer Medicine and School of Basic Medical Sciences, Southwest Medical University, Luzhou, Sichuan, 646000, China. E-mail:* [*hetao198@swmu.edu.cn*](mailto:hetao198@swmu.edu.cn)

*^b^State Key Laboratory of Biotherapy and Cancer Center, West China Hospital, and West China School of Basic Medical Sciences & Forensic Medicine, Sichuan University, and Collaborative Innovation Center for Biotherapy, Chengdu, 610041, China.*

*^c^Department of Biochemistry and Molecular Biology, Monash University, Clayton, VIC, 3800, Australia.*

*^d^The Second Affiliated Hospital of Chengdu Medical College, China National Nuclear Corporation 416 Hospital, Chengdu, Sichuan 610051, China. E-mail:* [*shaoji93@163.com*](mailto:shaoji93@163.com)

*^e^Key Laboratory of Molecular Biology for Infectious Diseases (Ministry of Education), Institute for Viral Hepatitis, Department of Infectious Diseases, the Second Affiliated Hospital, Chongqing Medical University, Chongqing, 400016, China*

*^1^These authors contributed equally to this work.*

*^*^ Corresponding authors.*


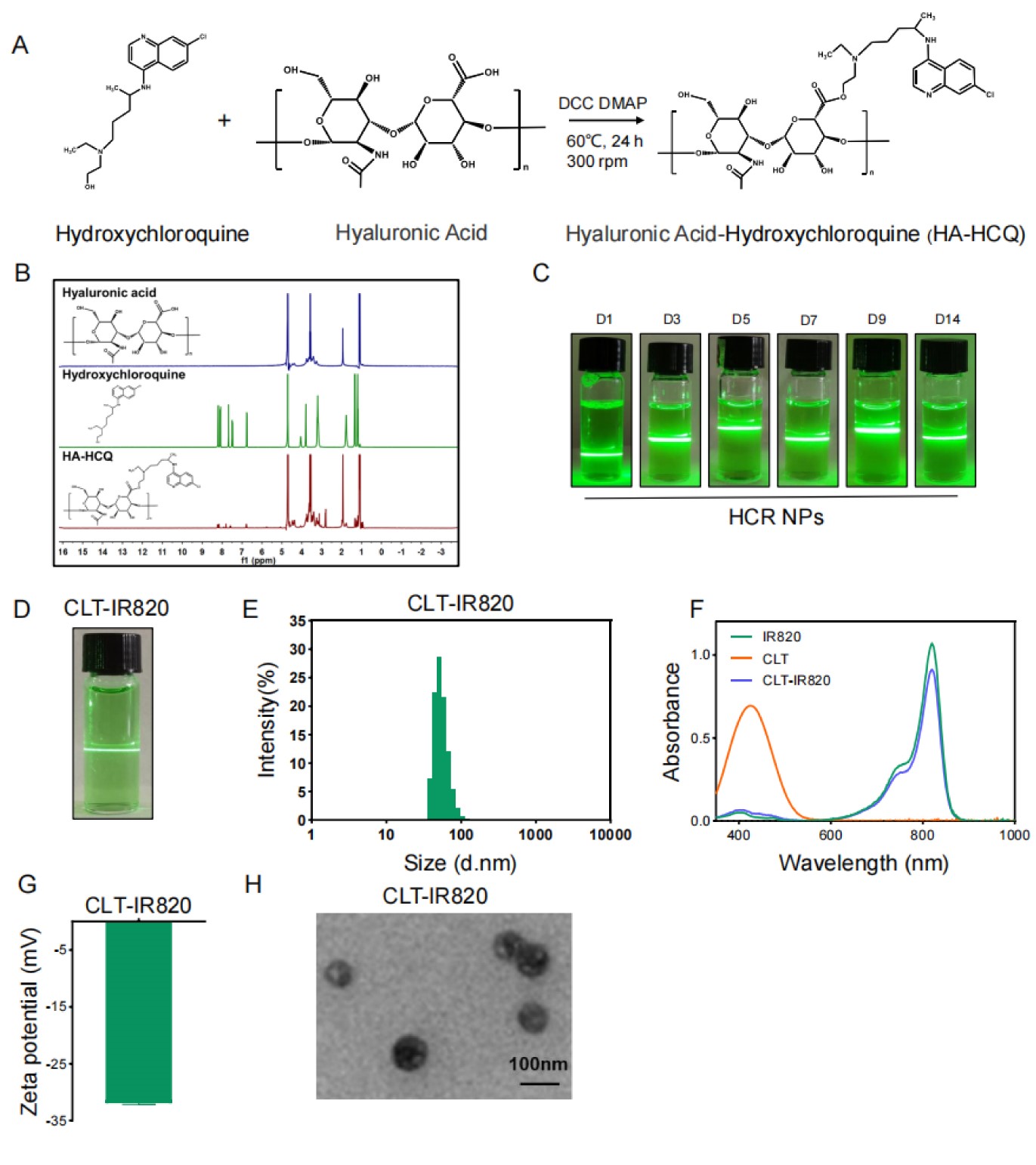


**Figure S1. Preparation and Characterization of CLT-IR820** **and stability of HCR NPs.** (A) Schematic illustration of the synthesis of HA-HCQ. (B) ^1^H NMR results of HA-HCQ. (C) The Tyndall effect of HCR NPs at the indicated time points. (D) The Tyndall effect of CLT-IR820. (E) Size distribution of CLT-IR820. (F) UV-vis absorption spectra of CLT-IR820. (G) Zeta potential of CLT-IR820. (H) TEM image of CLT-IR820. Scale bar: 100 nm.


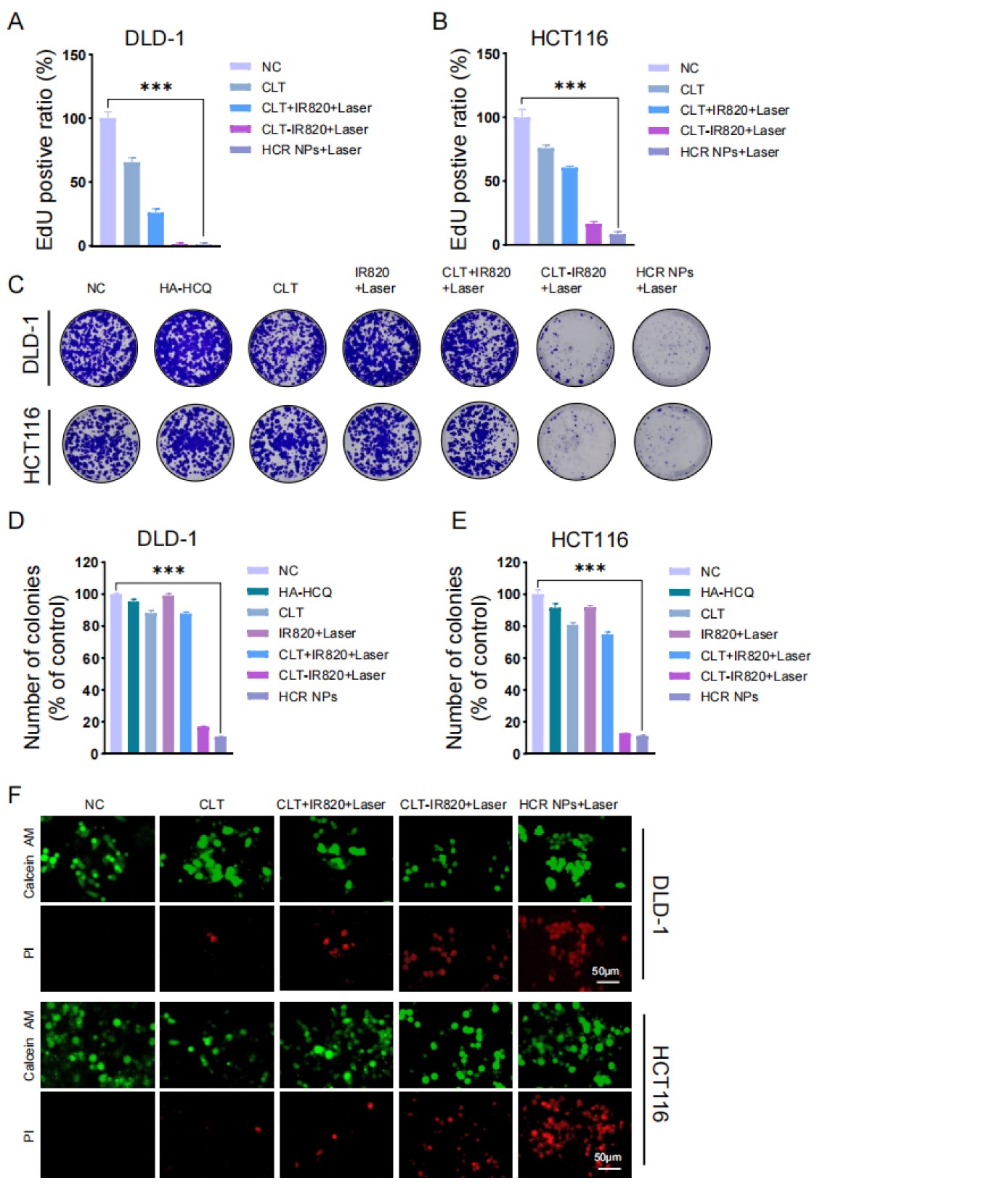


**Figure S2. Cytotoxicity of HCR NPs *in vitro*.** (A-B) Quantification of DLD-1 and HCT116 cells co-cultured with CLT, CLT+IR820+Laser, CLT-IR820+Laser, and HCR NPs+Laser in the EDU assay. (λ=808 nm, P =1.0 W/cm^2^; 40 s). ****P* < 0.001. (C-E) Representative images of the colony formation of DLD-1 and HCT116 cells treated with HA-HCQ, CLT, IR820+Laser, CLT+ IR820+Laser, CLT- IR820+Laser, and HCR NPs+Laser. (λ=808 nm, P =1 W/cm^2^; 60 s). ****P* < 0.001. (F) Cell imaging showing the survival of DLD-1 and HCT116 cells treated with CLT, CLT+ IR820+Laser, CLT- IR820+Laser, and HCR NPs+Laser. Live cells were marked with Calcein-AM (green fluorescence), while dead cells were marked with propidium iodide (red fluorescence). Scale bar: 50 µm. (λ=808 nm, P =1 W/cm^2^; 2 min).


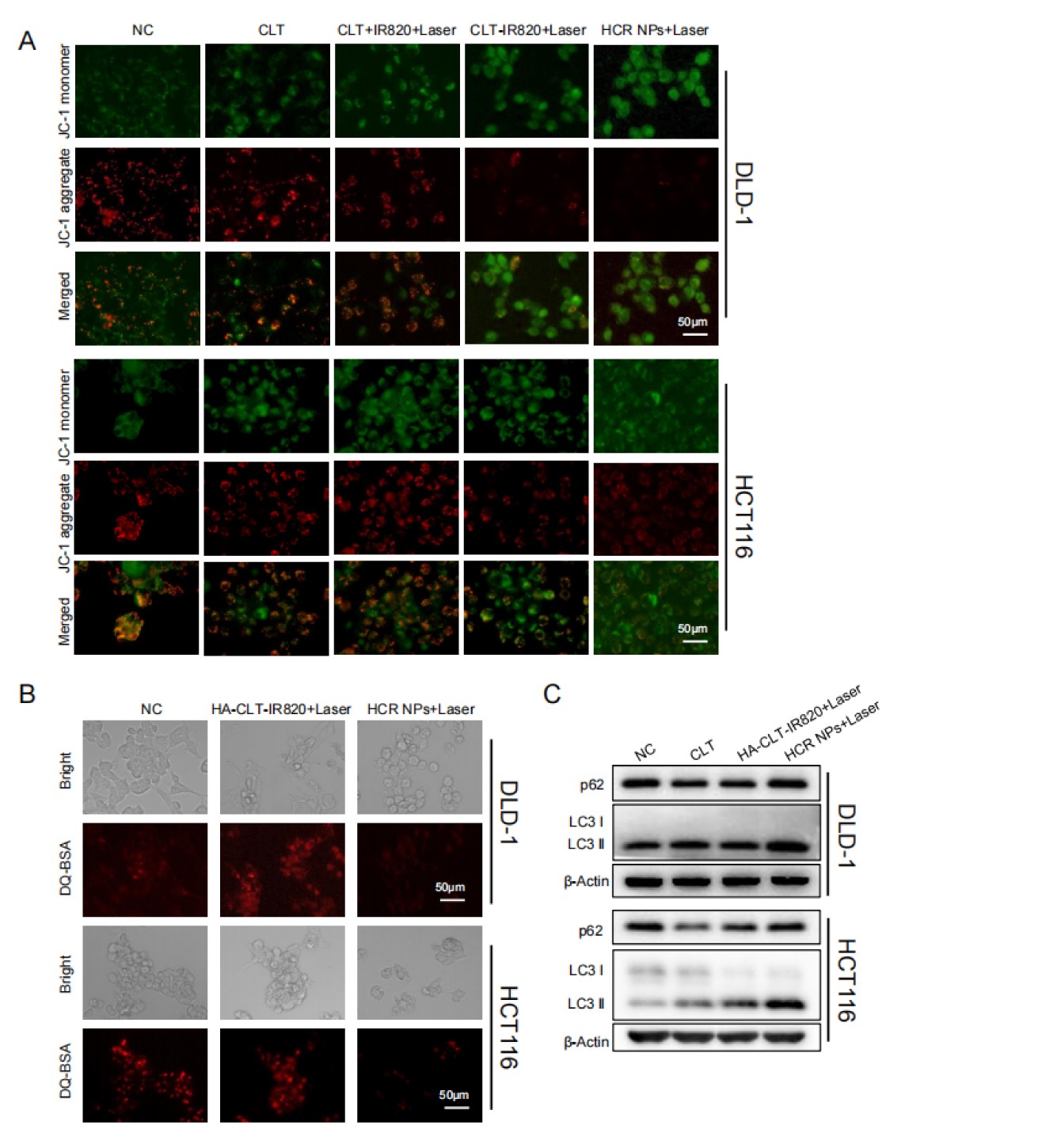


**Figure S3. Apoptosis and inhibition of autophagy.** (A) Fluorescent images of JC-1-stained DLD-1 and HCT116 cells, including CLT, CLT+IR820+Laser, CLT-IR820+Laser, and HCR NPs+Laser treatments. Scale bar: 50 μm. (λ=808 nm, P=1 W/cm^2^; 2 min). (B) Fluorescent images of DQBSA-stained DLD-1 and HCT116 cells, including HA-CLT-IR820, and HCR NPs treatments. Scale bar: 50 μm. (λ=808 nm, P=1 W/cm^2^; 2 min). (C) Immunoblot analysis of p62 and LC3-II in DLD-1 and HCT116 cells treated with CLT, HA-CLT-IR820, and HCR NPs. (λ=808 nm, P=1 W/cm^2^; 2 min).


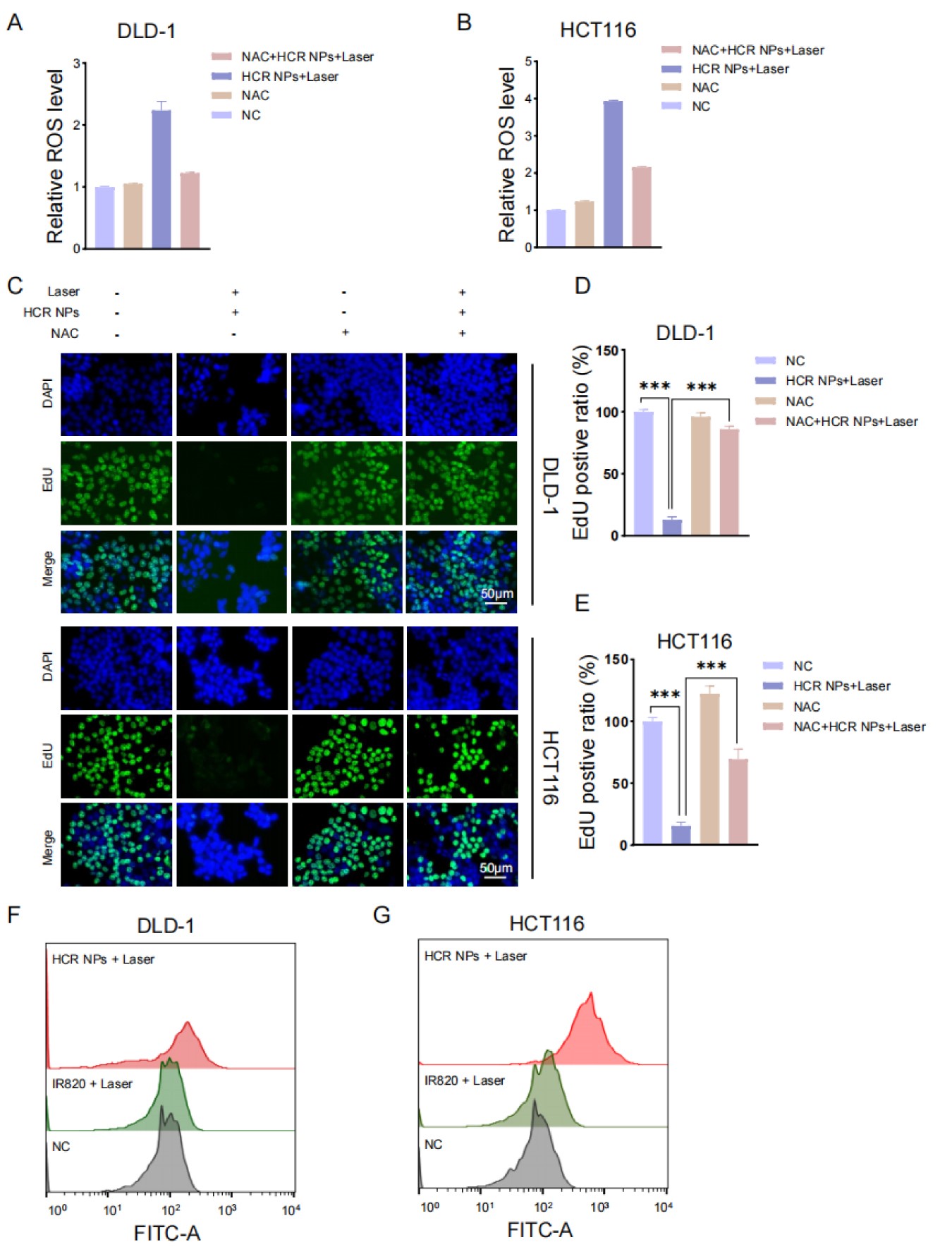


**Figure S4. HCR NPs-mediated ROS inhibited colorectal cancer cell survival.** (A-B) Flow cytometry statistical analysis of intracellular ROS generation in DLD-1 and HCT116 cells treated with HCR NPs+Laser with or without NAC treatment using DCFH-DA as a probe. (λ=808 nm, P=1 W/cm^2^; 2 min). (C-E) The proliferation of DLD-1 and HCT116 cells treated with HCR NPs+Laser with or without NAC treatment as measured by EdU assay. Scale bar: 50 μm. (λ=808 nm, P =1 W/cm^2^; 40 s). ****P* < 0.001. (F-G) Flow cytometry analysis of intracellular ROS generation in DLD-1 and HCT116 cells treated with IR820+Laser, or HCR NPs+Laser using DCFH-DA as a probe. (λ=808 nm, P=1 W/cm^2^; 2 min).


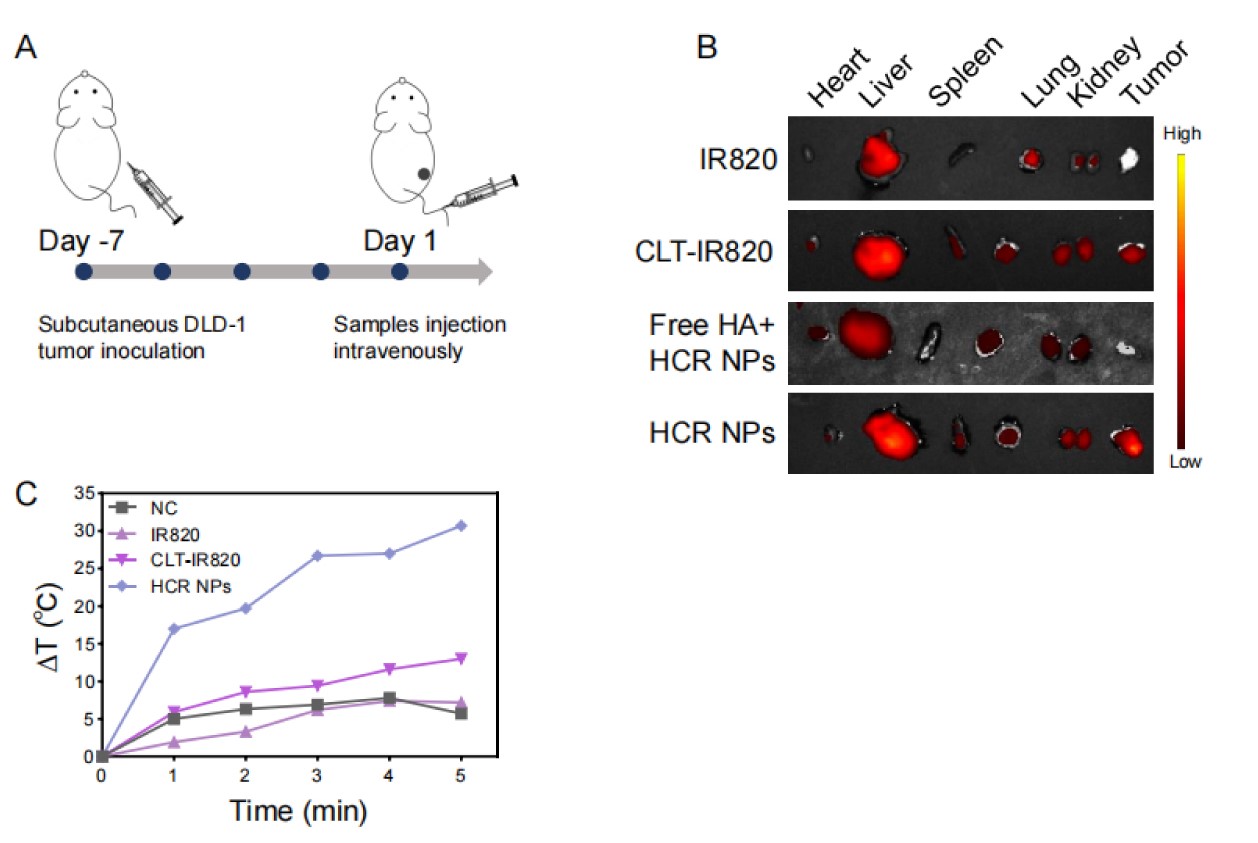


**Figure S5. The photothermal capacity of HCR NPs *in vivo.*** (A) Schematic illustration of the photothermal capacity of HCR NPs *in vivo*. (B) Fluorescence images of tumors and major organs at 24 h after injection with IR820, CLT-IR820, free HA+HCR NPs, and HCR NPs. (C) *In vivo* thermal analysis of different groups of treated mice. (λ=808 nm, P=1 W/cm^2^; 5 min).
